# Protease-activated receptor-1 (PAR1) promotes epithelial-endothelial transition through Twist1 in hepatocellular carcinoma

**DOI:** 10.1186/s13046-018-0858-4

**Published:** 2018-08-06

**Authors:** Ting Xiao, Qiang Zhang, Shumin Zong, Wei-long Zhong, Yuan Qin, Zhun Bi, Shuang Chen, Hui-juan Liu, Jun-jie Wei, Bi-jiao Zhou, Lu-meng Wang, Hong-gang Zhou, Yan-rong Liu, Tao Sun, Cheng Yang

**Affiliations:** 1 State Key Laboratory of Medicinal Chemical Biology and College of Pharmacy, Nankai University, No. 38 Tongyan Road, Haihe River Education Park, Jinnan District, Tianjin, 300350 China; 2 Tianjin Key Laboratory of Molecular Drug Research, Tianjin International Joint Academy of Biomedicine, No. 220 Dongting Road, Binhai District, Tianjin, 300457 China

**Keywords:** Protease-activated receptor-1, Vasculogenic mimicry, Twist1, Hepatocellular carcinoma, Epithelial–endothelial transition

## Abstract

**Background:**

Tumor cells transfer into endothelial cells by epithelial–endothelial transition (EET), which is characterized by vasculagenic mimicry (VM) in morphology. VM can change tumor microcirculation, progression, and metastasis. However, the molecular mechanisms of endothelial-like transition remain unclear. EET is a subtype of epithelial–mesenchymal transition (EMT). Twist1, a transcriptional regulatory factor of EMT, is an important factor that induces EET in hepatocellular carcinoma(HCC), but the upstream signal of Twist1 is unclear.

**Methods:**

Expression plasmids, Ca mobilization, and three-dimensional cultures were evaluated. Western blot assay, reporter gene assay, and immunofluorescence staining were conducted. A murine xenograft model was established. Analyses of immunohistochemistry, patient samples, and complementary DNA (cDNA) microarrays were also performed.

**Results:**

This study demonstrated that protease-activated receptor-1 (PAR1) can increase the expression of endothelial markers and enhance VM formation by upregulating Twist1 both in vitro and in vivo through thrombin binding. Thrombin not only activates PAR1 but also promotes PAR1 internalization in a time-dependent manner. Clinical pathological analysis further confirms that PAR1 expression is directly correlated with the endothelial marker expression, VM formation, and metastasis and indicates poor survival rate of patients with tumors.

**Conclusion:**

PAR1 promotes EET through Twist1 in HCC.

**Electronic supplementary material:**

The online version of this article (10.1186/s13046-018-0858-4) contains supplementary material, which is available to authorized users.

## Background

Tumor angiogenesis is a crucial step for tumor growth and is related to metastasis [1]. When a tumor enlarges to more than 1–2 mm in diameter, angiogenesis is essential to maintain its growth [1]. Angiogenesis is a relatively complex process, in which tumors cannot depend entirely on host endothelial cells to form blood vessels. Scholars have proposed several tumor angiogenesis pathways, such as vasculogenic mimicry (VM) and mosaic vessels [2, 3]. In 1999, Maniotis et al. [2] first reported VM based on the capability of highly aggressive melanoma cells to dedifferentiate into many cellular phenotypes, such as those with endothelial-like characteristics that can form vessel-like structures to provide blood supply. Clinical data review and meta-analysis revealed the direct correlation of tumor VM with poor prognosis of cancer patients [4–7]. However, the molecular mechanism of the VM transition of endothelial-like cell to tumor remains unclear.

Studies have reported the induction of VM by epithelial–mesenchymal transition (EMT) [8–10]. EMT-inducing transcription factor-Twist1 can promote the transcription of vascular endothelial (VE)-cadherin and induce the formation of VM channels [10]. However, the upstream signal of Twist1 is unclear. Epithelial–endothelial transition (EET), a subtype of EMT, produces endothelial-like phenotype of tumor cells, whereas endothelial-like phenotype can form VM to allow serum into tumor tissues [11]. Serum contains prothrombin, which is converted into thrombin via proteolytic cleavage in the tumor microenvironment [9, 12]. It was reported that thrombin, acting through protease-activated receptor-1 (PAR-1), promote EMT both in embryos development and colon cancer progression [13, 14]. We thus suppose that serum thrombin may induce VM through PAR1 activation. Therefore, the present study investigates the role of the thrombin/PAR1 pathway in the transition of tumor cells into endothelial-like cells and the underlying molecular mechanism. We found that serum thrombin can increase the expression levels of endothelial markers, such as VEGFR1, VEGFR2, and VE-cadherin, and enhance the formation of functional tubes in hepatocellular carcinoma (HCC). We showed that thrombin-activated PAR1 can increase the Twist1 transcription activity in vitro *and* in vivo, thereby increasing the expression of endothelial markers and facilitating VM formation. Clinical pathological studies further demonstrate the close relationship of PAR1 to VM, metastasis, and prognosis. Hence, PAR1 may be a potential target for future anticancer therapies.

## Methods

### Cell culture and transfection

HCC cell lines, namely, PLC-PRF-5, HepG2, SMMC7721, and HepG2/M (HepG2 high metastasis subclone), were obtained from Nanjing Keygen Cell Bank (Nanjing, China). All cell lines were authenticated by short tandem repeat analysis. The cells were cultured in RPMI 1640 or DMEM supplemented with 10% fetal bovine serum (Hyclone, Logan, Utah, USA) (Additional file 1: Table S1). The vectors were transfected into the cells with Roche transfection reagents.

### Expression plasmids

PAR1 complementary DNA (cDNA) was synthesized (Genecopoeia, Rockville, USA), digested with EcoRI/XhoI, and cloned into the pcDNA3.1 vector. The plasmid pcDNA3-Twist1-Flag was constructed using standard molecular cloning techniques. The constructs were checked by DNA sequencing. Small interfering RNAs (siRNAs) against human PAR1 and Twist1 were designed and verified to be specific to these proteins. The PAR1 siRNA sequence was 5′-AAGGCUACUAUGCCUACUACU-3′. The Twist1 siRNA sequence was 5′-AAGCTGAGCAAGATTCAGACC-3′. The U6 promoter with a PAR1 or Twist1 siRNA insert was cloned into pRNA-U6-Neo (Genscript, Piscataway, New Jersey, USA). A nonsilencing siRNA sequence (target sequence: 5′-AATTCTCCGAACGTGTCACGT-3′) was used as negative control.

### Ca mobilization

Cells were plated in a 384-well plate and incubated in 5% CO_2_ atmosphere at 37 °C overnight. FLIPR Calcium Evaluation Kit 5 (Molecular Devices, San Francisco, California, USA) was used to measure changes in intracellular Ca levels. Prior to the measurement, the cells were added with loading dye from the kit and incubated at 37 °C for 2 h at 37 °C. The plates were placed at room temperature until the assay was performed. The plates were directly transferred to the FLIPR instrument for Ca testing.

### Three-dimensional cultures

Tumor cells were suspended in medium and tiled on Matrigel (BD, Franklin lake, New Jersey, USA). The tumor cells were incubated at 37 °C for 24 h. VM tubes were captured by an inverted microscope.

### Western blot assay

Proteins from cell lysates were separated by SDS–PAGE and transferred onto membranes, which were tested with various antibodies (Additional file 1: Table S2). Blots were developed using an enhanced chemiluminescence detection kit (Millipore, Massachusetts, USA). Glyceraldehyde-3-phosphate dehydrogenase(GAPDH) was used as internal loading control.

### Reporter gene assay

AP1, STAT3, NF-κB, and MYC promoters were cloned into the pGL6-TA luciferase reporter vector (Additional file 1: Table S3). Twist1, Twist2, Snail1, Slug, VEGFR1, VEGFR2, and VE-cadherin promoters were purchased from Genecopoeia (Rockville, USA) and were cloned into the pEZX-PG04 luciferase reporter vector (Additional file 1: Table S4). Transactivation assays were performed with the Dual-luciferase Reporter Assay System (Mlbio, Shanghai, China) and measured with the Luminoskan Ascent Reader System (Thermo, Massachusetts, USA).

### Immunofluorescence staining

After transfection, tumor cells were grown on glass slides overnight and fixed in ice-cold methanol. Primary antibodies against PAR1, Twist1 were used at 1:50 dilution. FITC and TRITC fluorescein-conjugated secondary antibodies were used as labels for the immunofluorescence assay. DAPI was used to stain the nuclei, and the samples were imaged by fluorescence microscopy (Nikon, Japan).

### Murine xenograft model

Six-week-old female NIH BALB/c-null mice were kept in specific pathogen-free animal facilities (SPF) of the Tianjin International Joint Academy of Biomedicine. Each group consists of five mice. After transfection, 1 × 10^6^ PLC-PRF-5 cells were subcutaneously injected into the back of each mouse. After 25 days, the experiments were terminated. Tumors were harvested and fixed in 10% formaldehyde solution for subsequent tests.

### Immunohistochemistry

The tissue sections were pretreated in a microwave, blocked, incubated using a series of antibodies, and stained with DAB and hematoxylin. The results were captured with a microscope (Olympus, Japan). Negative controls were prepared using PBS in lieu of the first antibody. The sections were read by two separate pathologists without any knowledge on the clinical pathology data of each patient. Both intensity and percentage of the positive cells were determined and multiplied (staining index). A staining index of > 6 was defined as high expression, whereas staining index of < 6 was considered low expression [15].

### Patient samples

HCC tissue microarrays containing 96 samples were obtained from US Biomax (Shanghai, China) for IHC analysis. Two certified pathologists individually examined the tissue blocks according to the WHO’s published standards of diagnosis, classification, and pathological grade. Consents from both the hospital and patients were secured prior to obtaining specimens. All tissues were collected under the highest ethical standards, in which the donors were completely informed on and agreed upon. All human tissues were collected following HIPPA approved protocols.

### cDNA microarrays

PLC-PRF-5 cells were seeded in 60 mm dishes to 70–80% confluence, transfected with PAR1 or Twist1, and stimulated with or without 1 U/ml thrombin. The samples were sent to Genergy Biotechnology (Shanghai, China) for RNA detection. Genes in the experimental groups expressing proteins (higher in amount by twofold compared with that of the control group) showed significantly different expression profiles.

### Statistical analysis

All data were evaluated using SPSS v.17.0. Differences were considered significant at *P* < 0.05.

## Results

### Expression level of PAR1 activated by thrombin is correlated with endothelial-like transition in HCC cells

**Fig. 1:**
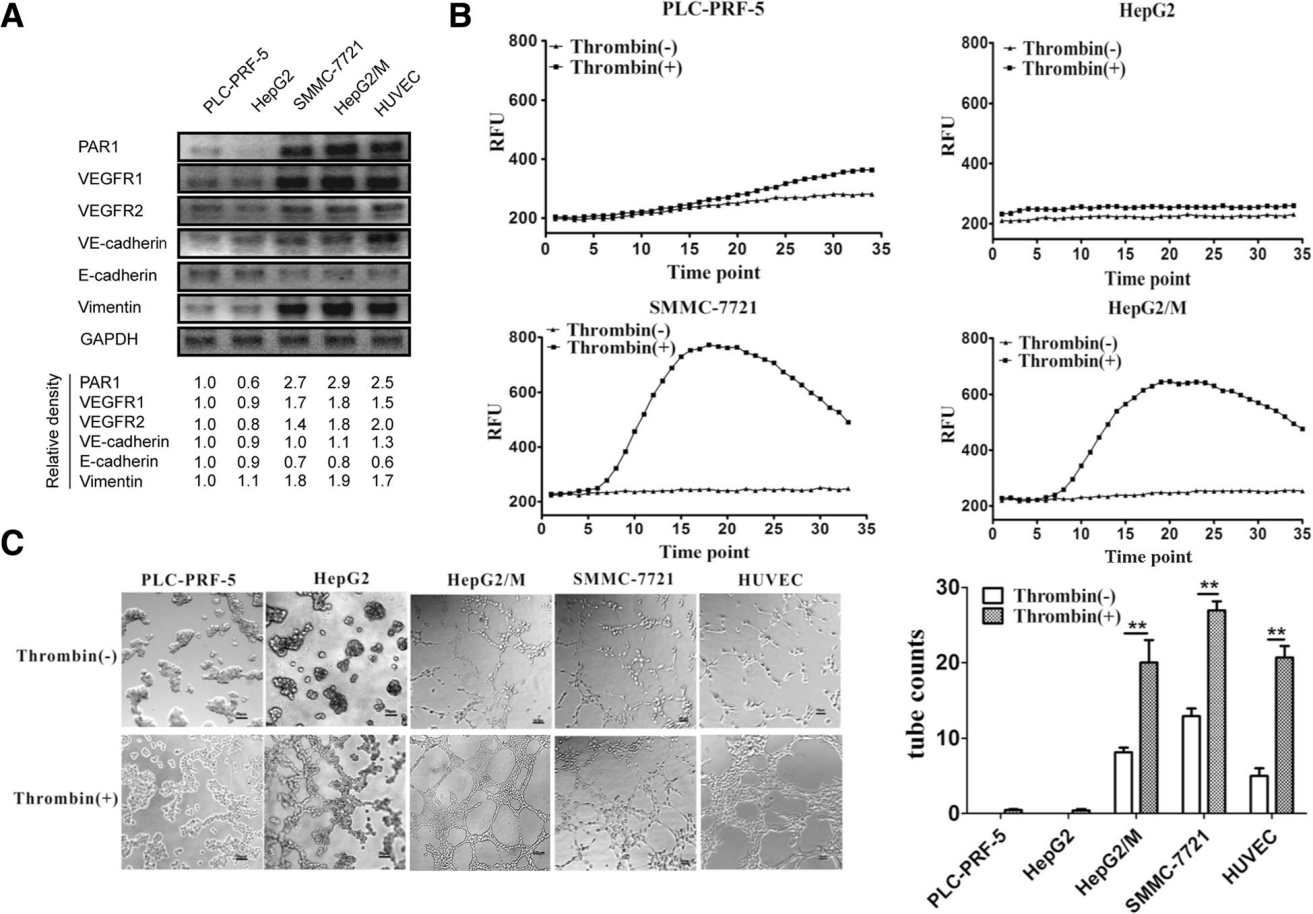
Expression level of PAR1 when activated by thrombin is correlated with endothelial-like transition in HCC cells. **a** Western blot assay of PAR1, endothelial, and EMT markers in different HCC cell lines and HUVEC. The endothelial cell line HUVEC has high expression of PAR1, endothelial, and EMT markers. Similar with HUVEC, SMMC-7721 and HepG2/M cells highly expressing PAR1 have higher expression levels of endothelial and mesenchymal markers than PLC-PRF-5 and HepG2 cells, which have low PAR1 expression. **b** Ca flux assays used to evaluate PAR1 activation upon thrombin binding in different HCC cell lines. (RFU: relative fluorescent unit; the higher RFU reflect, the higher the Ca ion concentration) (**c**) In vitro assay of VM tube formation in 3D gel at 20 h with or without 1 U/ml thrombin. HepG2/M and SMMC-7721 cells can form more VM tubes than PLC-PRF-5 and HepG2 cells. After thrombin stimulation, HepG2/M and 7721 cells produced twofold more VM structures than cells without thrombin (mean ± sd; *n* = 3 in triplicate; *P < 0.05, **P < 0.01)

Analysis of the western blot indicated the expression levels of the PAR1 protein in various HCC cell lines. SMMC-7721 and HepG2/M cells exhibited higher PAR1 expression than PLC-PRF-5 and HepG2 cells. PAR1 expression level was positively correlated with the expression levels of endothelial (VEGFR1, VEGFR2, and VE-cadherin) and mesenchymal (Vimentin) markers and negatively correlated with epithelial markers (E-cadherin) (Fig. 1a). When Ca flux was tested in these HCC cells, thrombin-activated PAR1 led to an increase in the intracellular Ca flux. SMMC-7721 and HepG2/M cells with high PAR1 expression were induced by thrombin toward the high Ca influx into the cytoplasm. Meanwhile, PLC-PRF-5 and HepG2 cells with low PAR1 expression showed minimal changes in Ca flux upon thrombin induction (Fig. 1b). The tube formation ability of these cells was assessed by three dimensional (3D) cultures on Matrigel. SMMC-7721 and HepG2/M cells expressing high levels of PAR1 formed typical tube-like structures on the Matrigel upon thrombin induction. However, PLC-PRF-5 and HepG2 cells expressing low levels of PAR1 did not form typical tube-like structures even in the presence of thrombin (Fig. 1c).

### PAR1 promotes EET in both phenotype and function

**Fig. 2:**
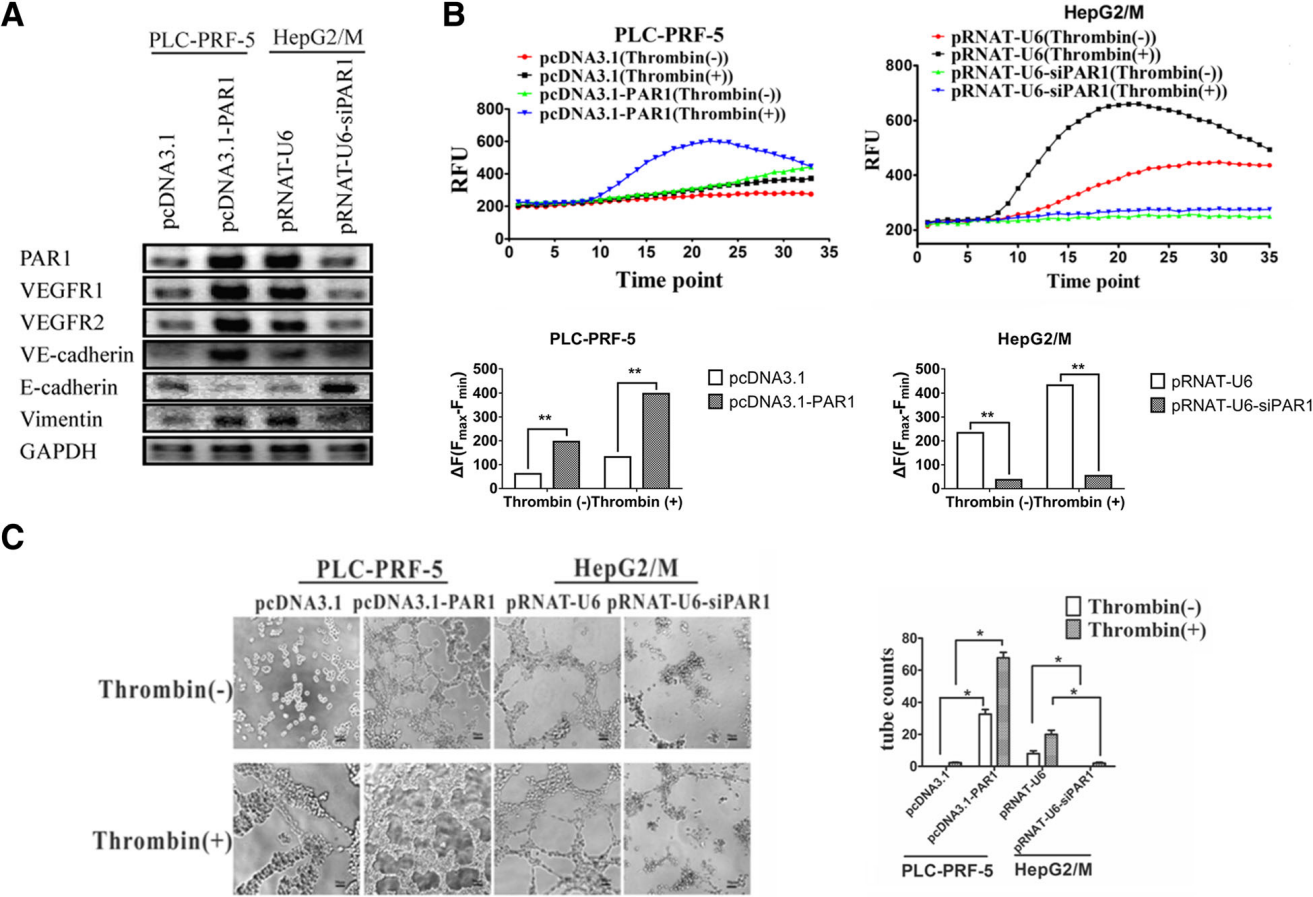
Functions of PAR1 in HCC cells when overexpressed or knocked down by siRNA. **a** Western blot assays of VEGFR1, VEGFR2, VE-cadherin, E-cadherin, and Vimentin in PLC-PRF-5 cells transfected with PAR1; HepG2/M cells transfected with PAR1 siRNA. **b** Ca flux changes in cells at different PAR1 levels with or without thrombin stimulation at 1 U/ml. In the presence of thrombin, Ca signals in PLC-PRF-5/PAR1 cells were significantly higher than in the absence of thrombin. When PAR1 in HepG2/M cells was knocked down by siRNA, Ca signals displayed a twofold decrease even with thrombin stimulation. **c** PAR1 expression levels also affected the VM tube formation in HCC cells, as assessed in 3D gel culture. PAR1 overexpression in PLC-PRF-5 cells can increase VM tube formation, and PAR1 knockdown can decrease VM tube formation in HepG2/M cells with and without thrombin stimulation. (mean ± sd; *n* = 3 in triplicate; *P < 0.05, **P < 0.01)

HepG2/M cells expressing high PAR1 level and PLC-PRF-5 expressing low PAR1 level were used to study the role of PAR1 in EET. The PLC-PRF-5 cell model overexpressing PAR1 and the HepG2/M cell model with knocked down PAR1 were established before the expression levels of endothelial, epithelial, and mesenchymal markers were measured in response to changes in PAR1 expression and activation. Overexpression of PAR1 in PLC-PRF-5 cells upregulated VEGFR1, VEGFR2, VE-cadherin, and vimentin and downregulated E-cadherin (Fig. 2a), suggesting that the cells obtained endothelial phenotype and underwent EET. Moreover, knocking down of PAR1 by siRNA decreased the expression levels of endothelial and mesenchymal markers and increased the expression of E-cadherin. These results further confirm the correlation between PAR1 expression and the formation of endothelial phenotype of tumor cells.

Ca flux assay verified the effects of overexpression or knocking down of PAR1. Upon thrombin induction, the Ca flux in PLC-PAR1 cells significantly increased compared with that in PLC cells. Moreover, the Ca flux in HepG2/M-siPAR1 cells was significantly lower than that in HepG2/M cells (Fig. 2b). However, Ca flux, though relative low, was also detected in cells highly expressing PAR1 even without exogenous thrombin. We uppose that the fetal bovine serum in the cell culture system may contain certain levels of thrombin, which can activate PAR1. This hypothesis was tested using a culture system without serum and Ca flux was not detected after PAR1 overexpression in PLC cells (data not shown).

HepG2/M cells highly expressing PAR1 and PLC-PRF-5 cells lowly expressing PAR1 were incubated in Matrigel culture to examine the correlation between PAR1 and VM formation in vitro. PAR1 overexpression (Fig. 2c) and activation promoted the formation of tube-like structures. PAR1 knockdown reduced the formation of tube-like structures (Fig. 2c). Hence, PAR1 activation promoted in vitro VM formation.

### PAR1 promotes VM formation through the Twist1 pathway

**Fig. 3:**
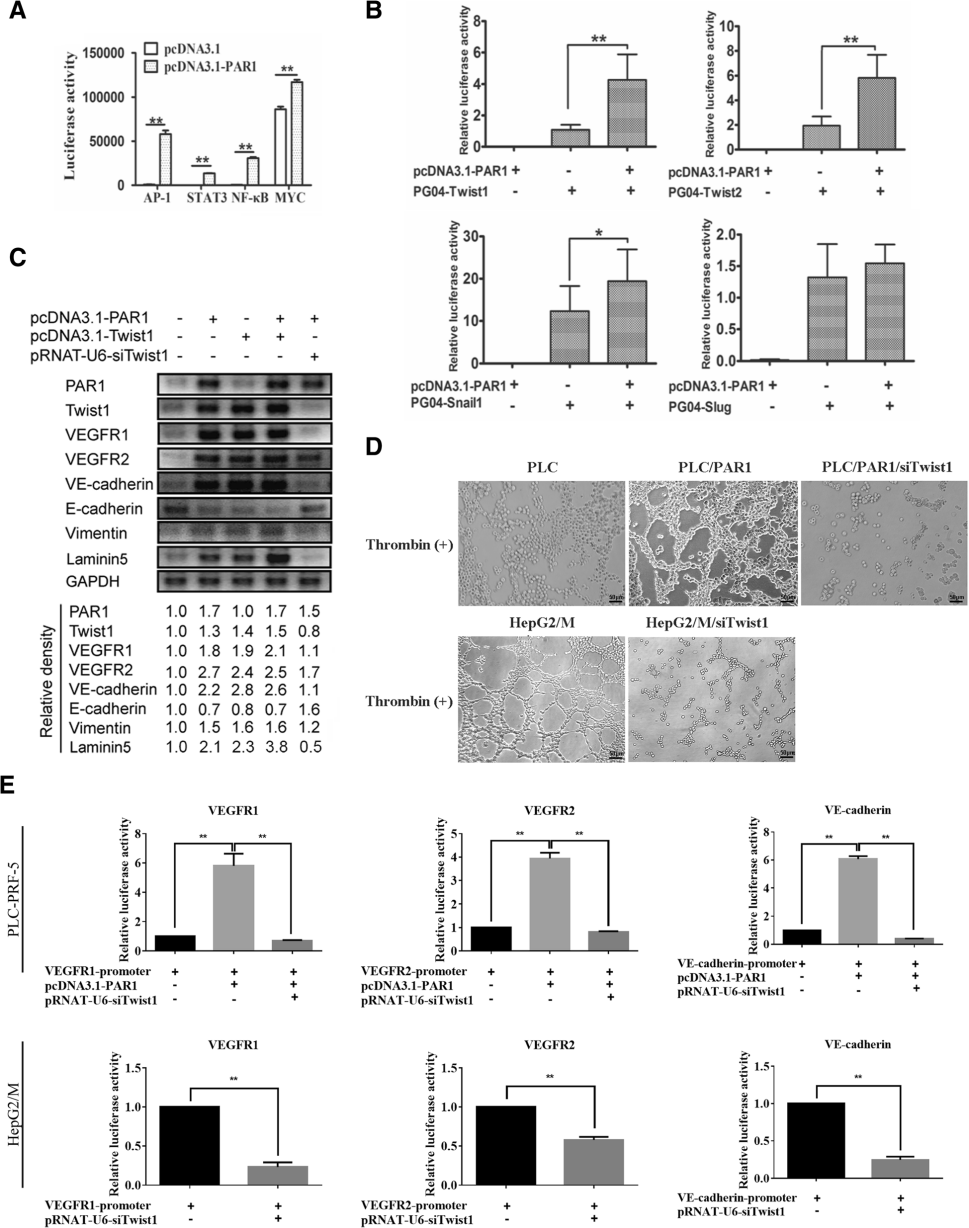
PAR1 promoted VM formation in vitro through Twist1. **a** and (**b**) The transcriptional regulatory activities of AP1, STAT3, NF-κB, and MYC (**a**) and Twist1, Twist2, Snail1 and Slug (**b**) were measured with the Dual-luciferase Reporter Assay and data was collected with the Luminoskan Ascent Reader System. PAR1 overexpression in PLC-PRF-5 cells significantly increased the transcriptional regulatory activities of AP1, STAT3, NF-κB, and MYC (**a**) and the transcriptional activities of Twist1, Twist2, Snail1 and Slug (**b**). **c** The expression levels of endothelial and mesenchymal markers of PLC-PRF-5 cells were examined by Western blot when transfected with vectors of pcDNA3.1-PAR1 or pcDNA3.1-Twist1, pcDNA3.1-PAR1/pcDNA3.1-Twist1 and pcDNA3.1-PAR1/pRNAT-U6-siTwist1, respectively. **d** The number of VM tubes in Matrigel was increased when PAR1 was overexpressed in PLC-PRF-5 cell lines and decreased after Twist1 was knocked down in HepG2/M- and PAR1 overexpressing PLC-PRF-5 cells. **e** PAR1 overexpression can increase the transcriptional activities of VEGFR1, VEGFR2, and VE-cadherin, while Twist1 knockdown decreased the promotional effects in PAR1 overexpressing PLC-PRF-5 cells and high PAR1-expressing HepG2/M cells. (mean ± sd; *n* = 3 in triplicate; *P < 0.05, **P < 0.01)

Luciferase reporter assay was conducted to check the activation of AP1, STAT3, NF-κB, and MYC in PLC-PAR1 compared with that in control cells and determine the molecular mechanism of PAR1 in EET. Overexpressing PAR1 overexpression in PLC-PRF-5 cells significantly increased the transcriptional regulatory activities of AP1, STAT3, NF-κB, and MYC (Fig. 3a) and the transcriptional activities of Twist1, Twist2, and Snail (Fig. 3b). Hence, PAR1 activated multiple signaling pathways. PAR1-induced EET may involve a series of complex signaling pathways, including EMT-related pathways of Twist1.

Western blot analysis showed that coexpressing PAR1 and Twist1 in PLC-PRF-5 cells significantly increased the expression of endothelial markers, including VEGFR1, VEGFR2, VE-cadherin, and Laminin5 compared with those in the control or groups overexpressing PAR1 or Twist1 alone. However, the outcomes were opposite in the knockdown of Twist1 in PLC-PAR1 cells. Hence, the promotion of EET by PAR1 is dependent on the Twist1 pathway (Fig. 3c).

The formation of tube-like structures by tumor cells were also determined. Overexpression of PAR1 in PLC-PRF-5 cells led to higher levels of VM tubes than those in the control groups. Knockdown of Twist1 weakened the promotion of tube formation of PAR1. In HepG2 cells highrexpressing PAR1, tube formation was also inhibited by knockdown of Twist1 (Fig. 3d), suggetsing that PAR1 can promote VM tube formation through the Twist1 pathway. Luciferase reporter assays were used to determine the roles of PAR1 and Twist1 in the transcriptional activation of endothelial markers. Overexpression of PAR1 in PLC-PRF-5 cells remarkably increased VEGFR1, VEGFR2, and VE-cadherin reporter activity, whereas knocking down Twist1 remarkably decreased the promotion of transcriptional activation of these endothelial markers by PAR1. In addition, Twist1 knockdown significantly inhibited the transcriptional activation of VEGFR1, VEGFR2, and VE-cadherin in HepG2 cells highexpressing PAR1 (Fig. 3e). A previous study demonstrated that Twist1 plays an important role in regulating EET [10]. These results show that PAR1 may be the upstream of Twist1 and promotes EET through Twist1.

### Internalized PAR1 can assist the nuclear translocation of Twist1 and further induce many signal transduction pathways

**Fig. 4:**
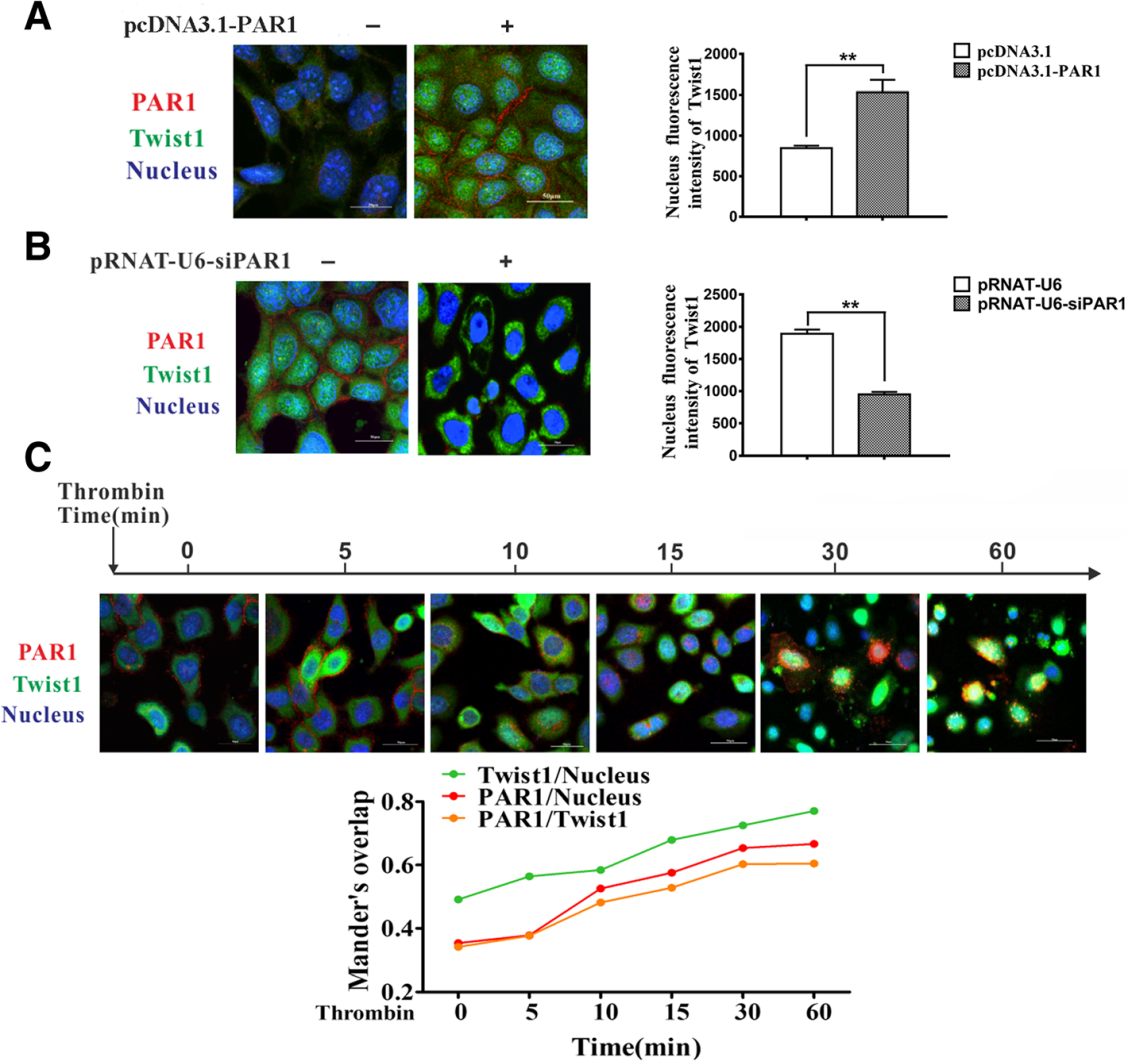
PAR1 internalization can promote Twist1 nuclear translocation. **a** Confocal imaging of PAR1 and Twist1 in PLC-PRF-5 cells transfected with PAR1 vectors. **b** Knockdown of PAR1 decreased Twist1 nuclear translocation in HepG2/M cells. **c** PAR1 internalization can occur after thrombin binding, as shown in the confocal microscopy images. Twist1 translocation to the nucleus was also increased. Mander’s overlap coefficient was used to quantify the degree of colocalization of both proteins (mean ± sd; *n* = 3 in triplicate; *P < 0.05, **P < 0.01)

Immunofluorescence was employed to evaluate the cellular location of Twist1 and PAR1 in tumor cells and determine how PAR1, as one of the G protein-coupled receptors (GPCRs) on the cell surface, promotes EET through Twist1. Overexpression of PAR1 assisted the nuclear translocation of Twist1 in PLC-PRF-5 cells (Fig. 4a), knocked down of PAR1 significantly reduced Twist1 nuclear expression in HepG2/M cells (Fig. 4b). In addition, thrombin-activated PAR1 induced the internalization of Twist1 in a time-dependent manner. After 60 min, Twist1 was completely internalized. PAR1 was also internalized into the nucleus over time (Fig. 4c). PAR1 may induce VM formation by internalization and increasing Twist1 nuclear translocation.

**Fig. 5:**
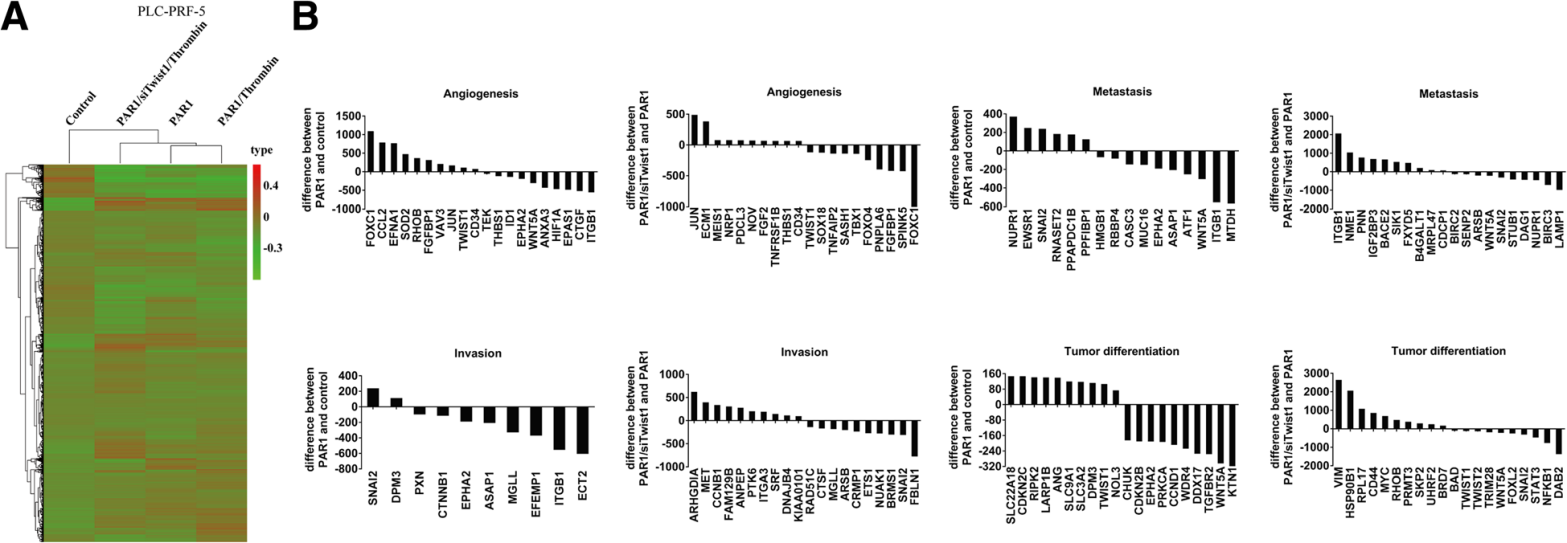
Omics analysis results of the gene expression profiles of PLC-control, PLC-PAR1, PLC-PAR1/thrombin and PLC-PAR1/siTwist1/Thrombin in PLC-PRF-5 cells. **a** Cluster analysis of mRNA data after normalization was shown as thermal map. The color changes refer the induction of gene expression. Lines indicate the clusters. PLC-PAR1/siTwist1/Thrombin and PLC-control were listed in the same cluster while PLC-PAR1 and PLC-PAR1/thrombin was located in another cluster. **b** Compared to PLC-control, PAR1 overexpression affects a series of genes participated in the angiogenesis, metastasis, invasion, and tumor differentiation functions. These functions can then be regulated by Twist1 knocking down after PAR1 overexpression

Gene expression profile array was used to examine the overall changes in the mRNA spectra of PLC-control, PLC-PAR1, PLC-PAR1/thrombin and PLC-PAR1/siTwist1/thrombin cell groups. Cluster and comparative analyses showed a distinct pattern of mRNA expression profile was found when Twist1 was knocked down after overexpressing PAR1 and adding thrombin (Fig. 5a). Thrombin-activated PAR1 can influence the expression levels of many genes related to angiogenesis, metastasis, invasion, and tumor differentiation. Hence, gene expression profiles induced by PAR1 were influenced by silencing Twist1 (Fig. 5b, Additional file 1: Table S5).

### PAR1/Twist1 pattern increases HCC growth, metastasis, and angiogenesis in the xenograft model

**Fig. 6:**
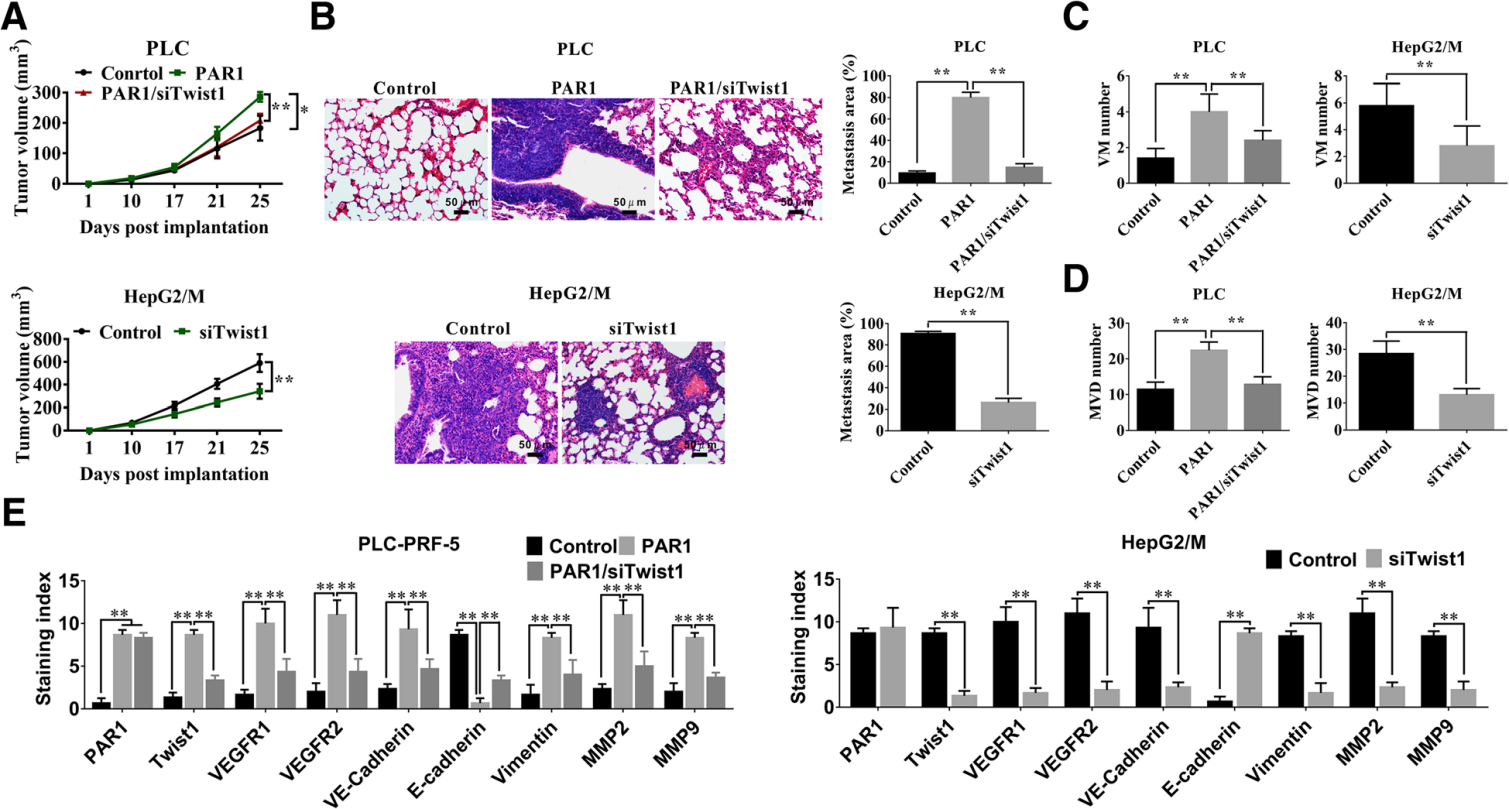
Over-expression of PAR1 promoted HCC growth and metastasis in the xenograft model. **a** Tumor growth in nude mice after implanting PLC-PRF-5 cells transfected with pcDNA3.1-PAR1 or pcDNA3.1-PAR1/pRNAT-U6-siTwist1 and HepG2/M cells transfected with pRNAT-U6-siTwist1 (*n* = 5 for each group). PLC-PAR1 transplanted tumors showed higher rate of tumor growth compared with the control group while PLC-PAR1/siTwist1 xenografts exhibited slower growth rate than PLC-PAR1 transplanted tumors. HepG2/M-siTwist1 xenografts grew slower than the HepG2/M transplanted tumors. **b** Overexpression of PAR1 promoted lung metastasis in PLC-PRF-5 xenograft model, and knocked down of Twist1 decreased the lung metastasis compared with PLC-PRF-5 and HepG2/M xenograft groups overexpressing PAR1. **c** Effects of PAR1 on VM formation in xenograft tissues. **d** Effects of PAR1 on MVD in xenograft tissues. **e** IHC results showed the effects of PAR1 on the expression of PAR1, Twist1, VEGFR1, VEGFR2, E-cadherin, or vimentin, MMP-2 and MMP-9. (mean ± sd; **P < 0.01)

The PLC-PRF-5 and HepG2/M xenograft models were used to examine the in vivo effects of PAR1 and Twist1 during tumor growth. Overexpression of PAR1 in the PLC-PRF-5 model significantly increased tumor growth and lung metastasis, whereas knockdown of Twist1 in HepG2/M or PLC-PRF-5 cells overexpressing PAR1 inhibited the promotional effects on tumor progression by PAR1 (Fig. 6a and b). VM formation and micro-vessel density (MVD) were determined in the excised tumors. Overexpressing PAR1 in the PLC-PRF-5 xenograft model significantly promoted VM formation and increased the MVD. When Twist1 was knocked down, the VM numbers and MVD induced by PAR1 decreased. In the HepG2/M xenograft model, Twist1 knockdown significantly inhibited VM formation and MVD (Fig. 6c and d, respectively).

Immunohistochemical assay of tumor tissues revealed that PLC-PAR1 and HepG2/M/PAR1 overexpressing tumors exhibited high expression levels of VEGFR1, VEGFR2, VE-cadherin, and vimentin and a low expression level of E-cadherin while Twist1 silence reversed the regulation effects of PAR1 (Fig. 6e). These results are consistent with the findings of the in vitro experiment.

### Expression levels of PAR1/Twist1 are correlated with angiogenesis in clinical data

**Fig. 7:**
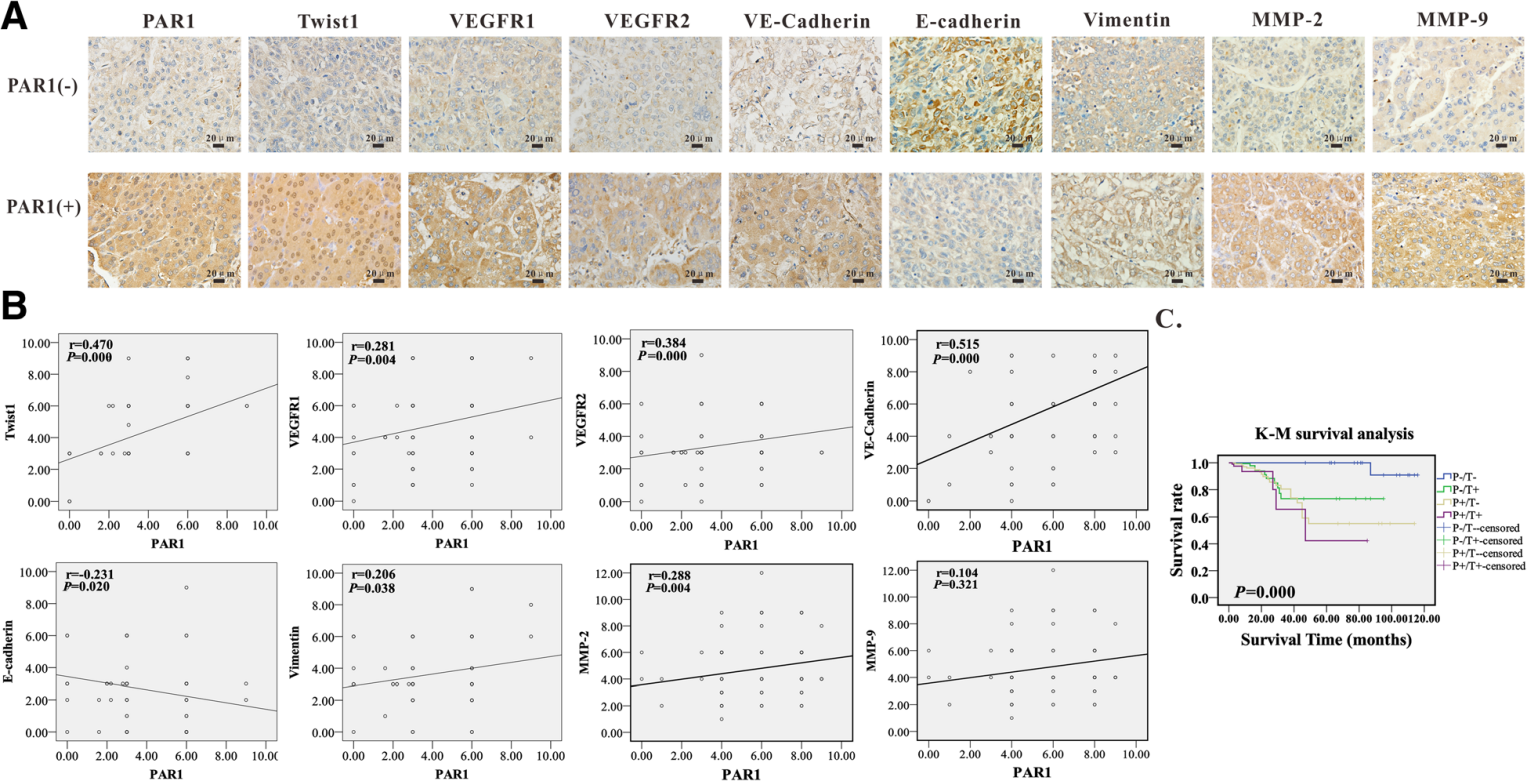
Clinical pathological analysis. **a** Representative section images of PAR1, Twist1, VEGFR1, VEGFR2, E-cadherin, vimentin, MMP-2 and MMP-9 in tissues where PAR1 was overexpressed and underexpressed (PAR1, Twist1, VEGFR1, VEGFR2, E-cadherin, or vimentin, MMP-2 and MMP-9) shown in brown; cell nucleus shown in blue). **b** The expression levels of Twist1, VEGFR1, VEGFR2, E-cadherin, vimentin, and PAR1 in human HCC tissues were scored by staining index and pearson correlation analysis was then performed. A positive correlation exists between PAR1, Twist1, VEGFR1, VEGFR2, vimentin, MMP-2 and MMP-9. A negative correlation exists between PAR1 and E-cadherin (*P* < 0.05). **c** Survival data of clinical cases. Survival rate of tumor patients was significantly decreased under high expression levels of both PAR1 and Twist1

The expression levels of PAR1 and Twist1 in 96 HCC cases were analyzed against the detailed clinical and pathologic information. PAR1 expression was correlated with tumor size, clinical stage, and pathological grade (Additional file 1: Table S6–8). Based on the comparisons between VM and non-VM groups and between low and high MVD groups, the results indicated the correlation of PAR1 expression with VM formation or MVD (Table 1). A further study was conducted to establish the relationship between PAR1 and VM-related proteins, such as VEGFR1, VEGFR2, VE-cadherin, E-cadherin, vimentin, MMP2, and MMP9. Significant correlations were found between the expression levels of these proteins and PAR1 in clinical liver cancer tissues (Fig. 7a and b, Additional file 1: Table S9).

**Table 1:** Relationship between PAR1/Twist1 expression and VM number or MVD

Variant	P^−^/T^−^	P^+^/T^−^	P^−^/T^+^	P^+^/T^+^	χ^2^	*P*
VM						
Negative	22	8	3	36	66.604	0.000**
Positive	1	3	5	18		
MVD						
< 230	23	7	8	0	90.146	0.000**
> 230	0	4	0	54		

** *P*<0.01

Kaplan–Meier survival analysis also suggested that the co-expression of PAR1 and Twist1 was correlated with poor survival of the patients. The survival time was also shorter than that in the negative group (Fig. 7c, Table 2).

**Table 2:** Multivariate analyses of factors influencing survival

Factor	Cox proportional hazards analysis		
	Relative Risk	95%CI	*P*
gender	1.107	0.450–2.725	0.825
age	1.009	0.539–1.890	0.977
Histological differentiation (I:II:III)	0.237	0.070–0.799	0.020*
Clinical stage (I:II:III: IV)	1.832	1.062–3.160	0.030*
PAR1/Twist1 (P^−^T^−^:P^+^T^−^:P^−^T^+^:P^+^T^+^)	1.519	1.110–2.077	0.009**

*Abbreviation*: *CI* confidence interval

*P < 0.05, ** *P* < 0.01

## Discussion

Highly aggressive tumor cells similar to a pluripotent and embryonic-like stem cell generally exhibit high plasticity and participate in VM formation and neovascularization [16, 17]. During VM tube formation, tumor cells acquire EC-like phenotypes, such as high expression levels of VEGFR1, VEGFR2, and VE-cadherin. This process is called EET [11]. Hypoxia, tumor interstitial fluid pressure (IFP), and angiogenesis cytokines are the main factors that induce EET [18, 19]. However, hypoxia- and IFP-induced neovascularization can also restore the local O and interstitial fluid levels in tumor tissues and eventually counteract high metastasis rate and poor survival of VM-positive patients. In addition, the levels of angiogenesis cytokines, such as VEGF, are relatively low during neovascularization. Therefore, the involvement of changes in the sensory receptor of the microenviroment in EET remains unknown.

Many studies have shown that the GPCR PAR1 has an important role in angiogenesis and thrombosis and in several tumor-promoting processes, including proliferation, invasion, and metastasis [10]. However, no data are available on the role of PAR1 in promoting tumor cell to acquire EC-like phenotype. The present study found that cells highly expressing PAR1 can increase VM tube formation, implying that PAR1 expression is related to tumor vascularization. When induced by thrombin, these cells can increase the expression levels of endothelial markers, such as VEGFR1, VEGFR2, and VE-cadherin, and repress the expression of the epithelial marker E-cadherin. Moreover, PAR1 activation by thrombin enhances ECM remodeling and VM tube formation. Hence, PAR1 promotes EET and VM formation.

Previous studies revealed that EET is a subtype of EMT [19]. As an important EMT transcription factor, Twist1 is also crucial for EET [11]. The present study demonstrated that PAR1 increases the activity of Twist1 promoters, suggesting the collaboration of PAR1 and Twist1 in promoting EET.

Ectopic expression or siRNA knockdown methods were used to show that EET promotion by PAR1 is synergized with Twist1. Hence, PAR1 may play a direct role in the upstream of Twist1, such as in the expression of endothelial markers, vascular function, ECM remodeling, and expression profiles. These results are consistent with the previous studies that activated PAR1 by thrombin induced EMT both in embryonic development and cancer progression [13, 14]. Fluorescence time-series analysis demonstrated that high PAR1 expression promotes the nuclear translocation of Twist1 and increases transcriptional regulatory activity upon thrombin induction. Moreover, thrombin-activation of PAR1 leads to PAR1 internalization, thereby increasing the occurrence of EET. Malignant tumors often exhibit hypercoagulability, which is correlated with high levels of activated thrombin [20]. The clinical pathological analysis of 96 patients with HCC further confirm that the expression levels of Twist 1 and endothelial markers in PAR1-positive HCC patients are higher than those in PAR1-negative patients. The above resuts further confirm the regulation of PAR1 on Twist1 and EET.

## Conclusion

Thrombin/PAR1 activation promote EET through Twist1 and PAR1 may be a potential target for future anticancer therapies. This study is the first to propose that the activation of the thrombin/PAR1 pathway is an important initiating factor to mediate EET through Twist1 in HCC. It provides a basis for searching tumor metastasis-related clinical markers and in-depth analysis of tumor differentiation to develop antitumor vascular drugs in the future.

## Additional file

Additional file 1: **Table S1.** Overview of the cell lines. **Table S2.** Primary antibodies used for WB, IF and IHC. **Table S3**. AP-1, STAT3, NF-κB, MYC luciferase reporter gene vector. **Table S4.** Twist1, Twist2, SNAI1, SNAI2, VEGFR1, VEGFR2 and VE-cadherin promoter reporter clones. **Table S5.** Differential expression genes of expression profiles analysis. **Table S6.** Correlation between PAR1 and clinicopathologic characteristics of patients with HCC. **Table S7.** Correlation between Twist1 and clinicopathologic characteristics of patients with HCC. **Table S8.** Relationship between PAR1/Twist1 expression and clinicopathologic parameter. **Table S9.** Relationship between PAR1/Twist1 positive and VEGFR1, VEGFR2, E-cadherin and Vimentin expression. (DOCX 41 kb)

## Abbreviations

AP1: apetala1
EET: epithelial–endothelial transition
EMT: epithelial–mesenchymal transition
GAPDH: glyceraldehyde-3-phosphate dehydrogenase
GPCR: G protein-coupled receptor
HCC: hepatocellular carcinoma
MMP: matrix metalloproteinase protease
MYC: myelocytomatosis oncogene
NF-κB: nuclear transcription factor kappa B
PAR1: protease-activated receptor-1
siRNA: small interfering RNA
STAT3: signal transducers and activators of transcription 3
VE-cadherin: vascular endothelial cadherin
VEGF: vascular endothelial growth factor
VEGFR1: vascular endothelial growth factor receptor1
VEGFR2: vascular endothelial growth factor receptor2
VM: vasculargenic mimicry

## References

[1] Folkman J (1971). Tumor angiogenesis: therapeutic implications. N Engl J Med.

[2] Maniotis AJ (1999). Vascular channel formation by human melanoma cells in vivo and in vitro: vasculogenic mimicry. Am J Pathol.

[3] Zhang S, Zhang D, Sun B (2007). Vasculogenic mimicry: current status and future prospects. Cancer Lett.

[4] Cao Z (2013). Tumour vasculogenic mimicry is associated with poor prognosis of human cancer patients: a systemic review and meta-analysis. Eur J Cancer.

[5] Chen L (2015). Vasculogenic mimicry is a major feature and novel predictor of poor prognosis in patients with orbital rhabdomyosarcoma. Oncol Lett.

[6] Guo Q (2016). Association between tumor Vasculogenic mimicry and the poor prognosis of gastric Cancer in China: an updated systematic review and meta-analysis. Biomed Res Int.

[7] Liu R (2012). Vasculogenic mimicry is a marker of poor prognosis in prostate cancer. Cancer Biol Ther.

[8] Bagnato A, Rosano L (2007). Epithelial-mesenchymal transition in ovarian cancer progression: a crucial role for the endothelin axis. Cells Tissues Organs.

[9] Ma JL (2011). Role of twist in vasculogenic mimicry formation in hypoxic hepatocellular carcinoma cells in vitro. Biochem Biophys Res Commun.

[10] Sun T (2010). Expression and functional significance of Twist1 in hepatocellular carcinoma: its role in vasculogenic mimicry. Hepatology.

[11] Sun T (2011). Promotion of tumor cell metastasis and vasculogenic mimicry by way of transcription coactivation by Bcl-2 and Twist1: a study of hepatocellular carcinoma. Hepatology.

[12] Esmon CT, Owen WG, Jackson CM (1974). The conversion of prothrombin to thrombin. II. Differentiation between thrombin- and factor Xa-catalyzed proteolyses. J Biol Chem.

[13] Chang LH (2011). Thrombin induces expression of twist and cell motility via the hypoxia-inducible factor-1alpha translational pathway in colorectal cancer cells. J Cell Physiol.

[14] Archiniegas E (2004). Thrombin and its protease-activated receptor-1 (PAR1) participate in the endothelial-mesenchymal transdifferentiation process. DNA Cell Biol.

[15] Mattern J, Koomagi R, Volm M (1996). Association of vascular endothelial growth factor expression with intratumoral microvessel density and tumour cell proliferation in human epidermoid lung carcinoma. Br J Cancer.

[16] Hendrix MJ (2003). Molecular plasticity of human melanoma cells. Oncogene.

[17] Hendrix MJ (2003). Vasculogenic mimicry and tumour-cell plasticity: lessons from melanoma. Nat Rev Cancer.

[18] Du J (2014). Hypoxia promotes vasculogenic mimicry formation by inducing epithelial-mesenchymal transition in ovarian carcinoma. Gynecol Oncol.

[19] Sun B (2017). Epithelial-to-endothelial transition and cancer stem cells: two cornerstones of vasculogenic mimicry in malignant tumors. Oncotarget.

[20] Caine GJ (2002). The hypercoagulable state of malignancy: pathogenesis and current debate. Neoplasia.

