# Supplementary material for: Protease-activated receptor-1 (PAR1) promotes epithelial-endothelial transition through Twist1 in hepatocellular carcinoma
Source: J Exp Clin Cancer Res. 2018 Aug 6;37:185. doi: 10.1186/s13046-018-0858-4 (PMC6091192; doi:10.1186/s13046-018-0858-4)
Supplement: Supplementary file 1 — Table S1. Overview of the cell lines. Table S2. Primary antibodies used for WB, IF and IHC. Table S3. AP-1, STAT3, NF-κB, MYC luciferase reporter gene vector. Table S4. Twist1, Twist2, SNAI1, SNAI2, VEGFR1, VEGFR2 and VE-cadherin promoter reporter clones. Table S5. Differential expression genes of expression profiles analysis. Table S6. Correlation between PAR1 and clinicopathologic characteristics of patients with HCC. Table S7. Correlation between Twist1 and clinicopathologic characteristics of patients with HCC. Table S8. Relationship between PAR1/Twist1 expression and clinicopathologic parameter. Table S9. Relationship between PAR1/Twist1 positive and VEGFR1, VEGFR2, E-cadherin and Vimentin expression. (DOCX 41 kb) [file 13046_2018_858_MOESM1_ESM.docx]

Table S1. Overview of the cell lines

| **Cell lines** | **Source** | **Growth Properties** | **Morphology** | **Propagation** | | | **Subculturing** | **Preservation** | |
| --- | --- | --- | --- | --- | --- | --- | --- | --- | --- |
|  |  |  |  | **Medium** | **T** | **Atmosphere** |  | **Medium** | **T** |
| PLC | liver cancer | adherent | epithelial | RPMI1640+10%FBS | 37℃ | 95% air  5% CO_2_ | 1、remove medium  2、rinse with 0.25% trypsin, 0.03% EDTA solution  3、remove the solution and add an additional 1 to 2ml of trypsin-EDTA solution  4、allow the flask to sit at room temperature until the cells detach  5、add fresh culture medium, aspirate and dispense into new culture flasks  6、Subcultivation ratio: 1:3 to 1:5  7、Medium renewal: 2 to 3 times per week | Complete growth medium supplemented with 10% DMSO | liquid nitrogen vapor phase |
| SMMC-7721 | liver cancer | adherent | epithelial | DMEM (high glucose)+10%FBS |  |  |  |  |  |
| HepG-2 | liver cancer | adherent | epithelial | DMEM (high glucose)+10%FBS |  |  |  |  |  |
| HepG-2/M | liver cancer | adherent | epithelial | DMEM (high glucose)+10%FBS |  |  |  |  |  |

Table S2. Primary Antibodies used for WB, IF and IHC

| Antibody | Type | Company | Catalogue Number |
| --- | --- | --- | --- |
| PAR1 | Rabbit polyclonal | Abcam | ab32611 |
| Twist1 | Mouse monoclonal | Affinity | BF0018 |
| E-cadherin | Rabbit polyclonal | Affinity | AF0131 |
| VE-cadherin | Rabbit polyclonal | Affinity | AF6265 |
| VEGFR1 | Mouse polyclonal | Santa cruz | sc-271789 |
| VEGFR2 | Rabbit polyclonal | Affinity | AF6281 |
| Vimentin | Mouse monoclonal | Affinity | BF0071 |
| GAPDH | Mouse monoclonal | Affinity | T0004 |
| Laminin5 | Mouse polyclonal | Santa cruz | sc-13587 |

Table S3.AP-1, STAT3, NF-κB, MYC luciferase reporter gene vector

| **Reporter gene vector** | **Response element** | **Vector information** |
| --- | --- | --- |
| pAP1-TA-luc^*^ | AP1 response element 26-67  TGACTAATGACTAATGACTAATGACTAATGACTAATGACTAA | pGL6-TA |
| pSTAT3-TA-luc^*^ | STAT3 response element 32-86  TGCTTCCCG AACGTTGCTT CCCGAACGTT GCTTCCCGAA CGTTGCTTCC GAACGT | pGL6-TA |
| pNFκB-TA-luc^*^ | NFκB response element 26-65  GGGAATTTCCGGGAATTTCCGGGAATTTCCGGGAATTTCC | pGL6-TA |
| pMyc-TA-luc* | Myc response element 26-61  CACGTGCACGTGCACGTGCACGTGCACGTGCACGT | pGL6-TA |

* The reporter gene vector werepurchased from Beyotime Biotechnology (Shanghai, China)

Table S4. Twist1, Twist2, SNAI1, SNAI2, VEGFR1, VEGFR2 and VE-cadherin promoter reporter clones

| **Promoter reporter clones** | **Promoter sequence** | **Vector information** |
| --- | --- | --- |
| Twist1^*^ | \| Promoter Length:  1282 bp \| \| --- \| \| Sequence length upstream of TSS:  1043 bp \| \| Sequence length downstream of TSS:  238 bp \| | pEZX-PG04 |
| Twist2^*^ | \| Promoter Length:  1512 bp \| \| --- \| \| Sequence length upstream of TSS:  1416 bp \| \| Sequence length downstream of TSS:  95 bp \| | pEZX-PG04 |
| SNAI1^*^ | \| Promoter Length:  1255 bp \| \| --- \| \| Sequence length upstream of TSS:  1185 bp \| \| Sequence length downstream of TSS:  69 bp \| | pEZX-PG04 |
| SNAI2^*^ | \| Promoter Length:  1399 bp \| \| --- \| \| Sequence length upstream of TSS:  1235 bp \| \| Sequence length downstream of TSS:  163 bp \| | pEZX-PG04 |
| VEGFR1^*^ | \| Promoter Length:  1257 bp \| \| --- \| \| Sequence length upstream of TSS:  1093 bp \| \| Sequence length downstream of TSS:  163 bp \| | pEZX-PG04 |
| VEGFR2^*^ | \| Promoter Length:  1236 bp \| \| --- \| \| Sequence length upstream of TSS:  1046 bp \| \| Sequence length downstream of TSS:  189 bp \| | pEZX-PG04 |
| VE-cadherin | \| Promoter Length:  1231 bp \| \| --- \| \| Sequence length upstream of TSS:  1041 bp \| \| Sequence length downstream of TSS:  189 bp \| | pGL4.3 |

* The promoter reporter clones werepurchased fromGeneCopoeia（Guangzhou，China）

Table S5. Differential expression genes of expression profiles analysis

| Groups | | PAR1 VS control | PAR1/siTwist1 VS PAR1 |
| --- | --- | --- | --- |
| angiogenesis | upregulated genes | HSPB1 PTGS2 FOXC1 CCL2 EFNA1 SOD2 HYAL1 RHOB SPINK5 FGFBP1 VAV3 HYAL1 COL4A2 JUN ANG VAV3 TWIST1 SRPK2 AMOTL2 CD34 LEF1 F3 CD34 EGFL7 | ITGB1 ANXA2 ADM ITGB1 PTTG1 ITGB1 COL4A1 PTTG1 METAP2 TNFRSF12A NCL SHC1 BAIAP2L1 HYAL1 ANXA2 METAP1 JUN RHOB SGK1 WASF2 EPAS1 MET GPI SGK1 ECM1 PTPRM SERPINE1 ID1 ITGA5 ANPEP ID3 MCTS1 PRKCA B4GALT1 LEMD3 ITGA3 SIRT1 SRF ARHGEF4 RBCK1 LEMD3 NCL ANXA2 JMJD6 PTPN14 MEIS1 NRP1 PDCL3 NOV RBCK1 FGF2 TNFRSF1B THBS1 CD34 |
|  | downregulated genes | TEK HMGB1 FGF2 SERPINE1 CTNNB1 THBS1 ID1 EPHA2 PRKCA E2F7 ITGAV ROCK2 WNT5A CTGF TNFRSF12A DDAH1 ANXA3 ITGB1 HIF1A EPAS1 MTDH COL15A1 NCL CYR6 1ITGB1 CTGF | ADRB2 BRCA1 MCAM AAMP RAB13 F2R SASH1 TBX1 TWIST1 SOX18 TNFAIP2 TNFSF15 SASH1 MED28 ETS1 TBX1 WARS DDAH1 ZNF580 ITGAV CDK5 WNT5A FOXO4 ROCK2 WARS RAF1 HTRA1 ETS1 PNPLA6 FGFBP1 SPINK5 VEZF1 SOD2 VAV3 ATPIF1 COL15A1 CAV1 CCBE1 FOXC1 LAMA5 PTGS2 PTGS2 POFUT1 |
| invasion | upregulated genes | SNAI2 SNAI2 DPM3 | ITGB1 ITGB1 ITGB1 CCNB1IP1 ARHGDIA MET CCNB1 FAM129B ANPEP PTK6 ITGA3 SRF 11-Sep DNAJB4 KIAA0101 |
|  | downregulated genes | PXN CTNNB1 EFEMP1 EPHA2 ASAP1 MGLL ITGB1 EFEMP1 ECT2 ITGB1 | BIRC2 LPXN PPP1R12A PTPRK RAC1 DDR1 CTSB SH3PXD2A RAD51C SNAI2 MGLL ETS1 BIRC2 CTSF PDCD4 MGLL ARSB CRMP1 NUAK1 BRMS1 ETS1 FBLN1 SNAI2 NUAK1 BIRC3 CTNNA1 FBLN1 GOLPH3 FBLN1 CTNNA1 XRCC2 PDCD4 ACTN4 |
| metastasis | upregulated genes | NUPR1 SNAI2 EWSR1 RNASET2 PPAPDC1B PPFIBP1 SNAI2 | ITGB1 NME1 ITGB1 ITGB1 BACE2 PNN SIK1 IGF2BP3 FXYD5 BACE2 SIK1 LAMP2 LAMP2 B4GALT1 NME1 NME1 MRPL47 CDCP1 CDCP1 |
|  | downregulated genes | HMGB1 RBBP4 CASC3 MUC16 EPHA2 ASAP1 ATF1 WNT5A ITGB1 MTDH ITGB1 | MYLK MCAM BIRC2 B3GNT6 PTPRK SENP2 CTSB SNAI2 BIRC2 CTSF LAMP2 ZFYVE21 LAMP2 ARSB WNT5A CRMP1 LAMP2 NUAK1 RAF1 BRMS1 STUB1 ST6GALNAC6 DAG1 NUPR1 SPOP SNAI2 NUAK1 STUB1 BIRC3 LAMP1 CREB1 S100A4 S100A4 |
| tumor differentiation | upregulated genes | HSPB1 PTGS2 HIST1H1C CDKN1A KRT17 LMNA BCL2L13 KLF2 DDIT3 PSMD4 NUPR1 RHOB SNAI2 GAS6 NENF CDKN1C PDCD5 ITM2C GAS6 ZFP36 ELF3 MAGED2 ITM2C EZH2 COL4A2 MYCN STK3 KRT16 SLC22A18 CDKN2C RIPK2 LARP1B ANG SLC9A1 SLC3A2 IRF7 SNAI2 DPM3 TWIST1 RBM38 MAP1A USH1C IRF1 RNF19A CD34 LEF1 NOL3 IRF7 CD34 EGFL7 | NDRG1 GAPDH HSPD1 HSP90AA1 VIM NME1 VIM HSPD1 HSP90AA1 HSP90B1 PGK1 EEF1B2 HSP90AB1 LGALS1 FTH1 PLS3 PGK1 RPL17 PPP1CC DDIT3 DDX47 ANLN IGFBP3 COL4A1 CD44 EIF2A PNN SIK1 IGFBP7 INSIG1 TNFRSF12A NCL IGF2BP3 MYC EIF4A1 SHC1 MPZL2 STIP1 IGFBP3 RHOB PLS3 PPP2R5C PPP2R5C PDCD5 RBM15 RBM25 GPRC5A TNFRSF21 PRMT3 KPNA2 BNIP3L TPD52L2 TXNIP G3BP2 SKP2 BCCIP SIK1 WDR4 PRMT5 ANPEP AXIN1 ZAK AXL UHRF2 DDX47 TAF15 CDK6 EXOSC5 RBMX PRKCA CDCA8 CCND1 B4GALT1 PTK6 WNT10B NME1 NCAPG SIRT1 ACVR1B BNIP3L BCCIP BRD7 EXT2 EEF1B2 SYNCRIP TGFBR2 OAS3 JMJD1C CDCA2 PRDM4 BUB1B MAP1S FMNL2 G3BP2 TPD52 G3BP2 JMJD1C HNRNPC NCL SKP1 INCENP SRC MPZL2 RABEP1 TOP1 ATG7 EIF3B PPP1R8 RQCD1 CLTC JMJD6 NME1 EIF4E LEO1 PHF20 CDCP1 PTPN14 VPRBP NRP1 NOV HNRNPA1 FGF2 HNRNPA1 ADAM9 CAPRIN2 ERCC6L CD34 CDCP1 PBRM1 PTPN2 LGALS8 TAF4B |
|  | downregulated genes | HMGB1 UHRF2 PMEPA1 EIF4G2 GPRC5A C10orf54 FMNL2 TOP1 FGF2 G3BP2 RABEP1 ENAH BUB1B CTNNB1 TAF15 CADM1 PHF20 G3BP2 SMAD6 DDX47 OAS3 MPZL2 EFEMP1 TNFRSF21 CHUK CDKN2B EPHA2 PRKCA CCND1 LIMS1 WDR4 DDX17 TGFBR2 TGFBR2 G3BP2 WNT5A KTN1 TNFRSF12A TAF15 PATL1 PGK1 HIF1A ANLN HNRNPK PLS3 MTDH STK4 EFEMP1 NCL EEF1D ECT2 CYR61 HSPH1 NDRG1 CD44 MYC DKC1 MYC LIMS1 PGK1 HSP90AA1 | ANK3 FZD7 LARP1B KIF1B KAT2B RIPK1 MYLK C1QL4 USH1C ANK3 KITLG BRCA1 AHRR NCOA1 PVRL2 USP11 RNF19A BAX PEX1 BIRC2 RBM38 ZFPM1 RALGAPA1 IRF7 TRAF2 MEN1 NPNT AIFM1 RBM6 ATG4B BAD SASH1 LRRC16A TWIST1 LLGL1 KITLG CIAPIN1 EZH2 SLC3A2 ERF CD276 SMARCB1 STAT3 SENP2 DDR1 WDR44 TWIST2 CTSB FZD2 TNFAIP2 CIC CDKN2C PRKCH SLC22A18 SNAI2 BMPR1A SAV1 AGTPBP1 LUZP1 BRE CPNE1 PRAME KRT16 SASH1 MED28 BIRC2 PDCD4 SPNS1 TACSTD2 SEC31A TRIM28 TNKS1BP1 ARID1A RALY TAOK3 SLC9A1 ITM2C BRE DNAJB6 WNT5A TP53BP1 MAP3K8 EBAG9 FOXL2 EZH2 EIF4G1 EEF2K TEAD2 MYCN KRT18 CPNE1 RAF1 GAS6 PSMB8 STAT3 STUB1 LMO4 ITM2C HNRNPM GAS6 CSK FLNB FBLN1 SMAD5 XPC MCM3 UBR4 MFF NUPR1 KLF2 SNAI2 DAPK1 AGTPBP1 STUB1 CBX2 KRT17 HNRNPH1 SEC31A HIST1H1C TNS3 NBL1 YTHDF2 TFAP2A BCL2L13 CTNNA1 NFKB1 CAV1 CALM1 RNF114 FBLN1 HNRNPU NBL1 STAT3 BIN1 LRRFIP1 FBLN1 CTNNA1 PDCD4 CREB1 LMO4 BIN1 DAB2 KRT8 ALPL SLC7A5 ATP5A1 PTGS2 DAB2 DDIT4 MCM3 CARHSP1 MSLN OCIAD1 PTGS2 |

Table S6. Correlation Between PAR1 and Clinicopathologic Characteristics of Patients with HCC

| Variant | PAR1 | | χ^2^ | P |
| --- | --- | --- | --- | --- |
|  | Low | High |  |  |
| Age(year) |  |  |  |  |
| ＜45 | 30 | 18 | 8.181 | 0.008** |
| ≥45 | 16 | 32 |  |  |
| Sex |  |  |  |  |
| Female | 6 | 3 | 1.399 | 0.237 |
| Male | 40 | 47 |  |  |
| Histological differentiation |  |  |  |  |
| Ⅰ/Ⅰ~Ⅱ/Ⅱ | 38 | 40 | 0.107 | 0.798 |
| Ⅱ~Ⅲ/Ⅲ | 8 | 10 |  |  |
| Clinical stage |  |  |  |  |
| Ⅰ~Ⅱ | 34 | 26 | 4.909 | 0.027* |
| Ⅲ~Ⅳ | 12 | 24 |  |  |
| Tumor size |  |  |  |  |
| T1~T2 | 34 | 26 | 4.909 | 0.027* |
| T3~T4 | 12 | 24 |  |  |

*Significantly different.

Table S7. Correlation Between Twist1 and Clinicopathologic Characteristics of Patients with HCC

| Variant | Twist1 | | χ^2^ | P |
| --- | --- | --- | --- | --- |
|  | Low | High |  |  |
| Age(year) |  |  |  |  |
| ＜45 | 31 | 17 | 6.01 | 0.024* |
| ≥45 | 19 | 29 |  |  |
| Sex |  |  |  |  |
| Female | 5 | 4 | 0.048 | 0.827 |
| Male | 45 | 12 |  |  |
| Histological differentiation |  |  |  |  |
| Ⅰ/Ⅰ~Ⅱ/Ⅱ | 44 | 32 | 4.937 | 0.026* |
| Ⅱ~Ⅲ/Ⅲ | 6 | 14 |  |  |
| Clinical stage |  |  |  |  |
| Ⅰ~Ⅱ | 33 | 27 | 0.545 | 0.46 |
| Ⅲ~Ⅳ | 17 | 19 |  |  |
| Tumor size |  |  |  |  |
| T1~T2 | 33 | 27 | 0.545 | 0.46 |
| T3~T4 | 17 | 19 |  |  |

*Significantly different.

Table S8. Relationship between PAR1/Twist1 expression and clinicopathologic parameter

| Variant | P^-^/T^-^ | P^+^/T^-^ | P^-^/T^+^ | P^+^/T^+^ | χ^2^ | *P* |
| --- | --- | --- | --- | --- | --- | --- |
| Age(year) |  |  |  |  |  |  |
| ＜45 | 16 | 5 | 3 | 24 | 44.438 | 0.000** |
| ≥45 | 7 | 6 | 5 | 30 |  |  |
| Sex |  |  |  |  |  |  |
| Female | 2 | 2 | 1 | 4 | 130.333 | 0.000** |
| Male | 21 | 9 | 7 | 50 |  |  |
| Histological differentiation |  |  |  |  |  |  |
| Ⅰ/Ⅰ~Ⅱ/Ⅱ | 20 | 11 | 7 | 40 | 77.250 | 0.000** |
| Ⅱ~Ⅲ/Ⅲ | 3 | 0 | 1 | 14 |  |  |
| Clinical stage |  |  |  |  |  |  |
| Ⅰ~Ⅱ | 14 | 6 | 8 | 32 | 43.271 | 0.000** |
| Ⅲ~Ⅳ | 9 | 5 | 0 | 22 |  |  |
| Tumor size |  |  |  |  |  |  |
| T1~T2 | 14 | 6 | 8 | 32 | 43.271 | 0.000** |
| T3~T4 | 9 | 5 | 0 | 22 |  |  |
|  |  |  |  |  |  |  |

*Significantly different, *＜0.05, ** ＜0.01

Table S9. Relationship between PAR1/Twist1 positive and VEGFR1, VEGFR2, E-cadherin and Vimentin Expression

|  | P^-^/T^-^ | P^+^/T^-^ | P^-^/T^+^ | P^+^/T^+^ | χ^2^ | *P* |
| --- | --- | --- | --- | --- | --- | --- |
| VEGFR1 |  |  |  |  | 11.361 | 0.010** |
| low | 31 | 9 | 4 | 20 |  |  |
| high | 5 | 5 | 6 | 16 |  |  |
| VEGFR2 |  |  |  |  | 4.999 | 0.172 |
| low | 31 | 11 | 6 | 24 |  |  |
| high | 5 | 3 | 4 | 12 |  |  |
| E-cadheirin |  |  |  |  | 8.705 | 0.033* |
| low | 28 | 6 | 8 | 19 |  |  |
| high | 8 | 8 | 2 | 17 |  |  |
| Vimentin |  |  |  |  |  |  |
| low | 22 | 8 | 3 | 11 | 8.5 | 0.037* |
| high | 14 | 6 | 7 | 25 |  |  |

*Significantly different, *＜0.05, ** ＜0.01.
